# Nashville Medical Society

**Published:** 1859-02

**Authors:** 


### PROCEEDINGS OF THE NASHVILLE MEDICAL SOCIETY.

President A. H. Buchanan made a verbal report of interest and importance. He was lately called to see Mr. E., of this city, who had a violent bleeding of the nose, which alarmed him in no small degree, as his father had once nearly bled to death from the same cause. All the known remedies were had recourse to, but without avail. The Doctor then attempted compression. He was not aware that his remedy was a new one, but not knowing what to do in the case, and while reflecting on the matter, it appeared to him that he might stay this bleeding by simple compression. Sitting then before his patient, he put his thumb and finger on the carotid artery of the right side, and compressed it against the transverse processes of the cervical vertebræ. This stopped the bleeding almost in a moment. Twenty-five hours subsequently, it recommenced; the same practice was followed, and with the same immediate beneficial results. Since this the bleeding has not recurred. He therefore recommended compression of the carotid artery as a remedy for epistaxis.

The Doctor mentioned, also, two instances in which he had saved the lives of women by using compression. This was not original, as he had gained the idea from reading in a medical journal, he forgot which. In one case the woman was in parturition, the child was already born and hanging by the cord; the hemorrhage was awful; she was entirely exhausted, pale, almost dead. He relieved her of the placenta, and carrying up the finger, pressed the aorta, immediately above the bifurcation, against the spine, and the bleeding ceased readily, while an assistant swathed the legs in bandages.

Dr. J. F. May stated that he had once compressed the internal jugular vein, in a case in which the vein had been opened. A large, fibrous tumor was being dissected out, when numerous, deep adhesions in the substance of the neck were detected. Some of these were attached to the vein, and on traction being exercised the coat of the vein gave way. The hemorrhage was terrific. He compressed the vein at once against the processes of the vertebræ, and as the tumor was only two-thirds out, and he had not time to dissect it, he tore it out. Dr. Coolidge, of the U. S. N., assisted him in the operation, and kept the patient two hours and a half on the table, and by brandy, friction and blisters restored him. The vein was tied, and the man recovered, so that, in a fortnight after, the Doctor removed another tumor from the same subject. The cases on record of tying this vein are very few in number. Dr. Mott, of New York, reported a case some years ago, the first on record, he calls it; but Dr. May claims that his case preceded Dr. Mott's by several weeks. —*Nashville Journal.*

